# Accidental diode laser-induced full-thickness macular hole: a case report

**DOI:** 10.1186/s12886-025-03970-8

**Published:** 2025-03-17

**Authors:** Nazanin Zeinali Nezhad, Atiye Moradi, Shiva Pouradeli, Mohammad Rezaei Zadeh Rukerd, Mahdi Sharifzadeh Kermani

**Affiliations:** 1 Physiology Research Center, Institute of Neuropharmacology, Kerman University of Medical Sciences, Kerman, Iran; 2 Clinical Research Development Unit, Shafa Hospital, Kerman University of Medical Sciences, Kerman, Iran; 3 Department of Stem Cells and Developmental Biology, Cell Science Research Center, Royan Institute for Stem Cell Biology and Technology, ACECR, Tehran, Iran

**Keywords:** Diode laser, Retinal injury, Vitrectomy, OCT

## Abstract

Accidental ocular injuries caused by laser devices used in non-medical settings are rare but potentially vision-threatening. This case report describes a 24-year-old woman who sustained a full-thickness macular hole (FTMH) in the right eye following accidental diode laser exposure during a hair removal procedure at a beauty center. The injury occurred when the laser probe was inadvertently activated, striking the patient’s unprotected eye. The patient presented with profound visual loss in the affected eye, with visual acuity reduced to the level of hand motion. Comprehensive ophthalmological examination revealed a FTMH in the right eye, confirmed by optical coherence tomography (OCT), which showed complete disruption of the foveal retinal layers and cystic changes at the margins of the hole. The patient underwent surgical intervention with pars plana vitrectomy (PPV), internal limiting membrane (ILM) peeling, and gas tamponade to promote macular hole closure and restore retinal integrity. Despite successful anatomical closure of the macular hole, the patient’s visual prognosis remained guarded due to extensive photothermal damage to the retinal pigment epithelium and photoreceptor layers. This case underscores the devastating consequences of inadequate laser safety protocols in non-medical environments, the critical role of OCT in diagnosing and managing laser-induced retinal injuries, and the importance of timely surgical intervention.

## Introduction

Laser technology has become an indispensable tool across various medical and aesthetic fields due to its precision, versatility, and efficacy [1]. Among its many applications, diode lasers have gained widespread popularity in dermatological and cosmetic procedures, particularly for laser hair removal. Diode lasers operate at a wavelength of 800–810 nm in the near-infrared spectrum, targeting melanin in hair follicles to achieve selective photo thermolysis and permanent hair reduction. Their ability to penetrate deeply into the dermis while sparing surrounding tissues has made them a preferred choice for hair removal across a wide range of skin types, especially lighter-to-medium skin tones [2, 3]. However, despite their safety and effectiveness when used appropriately, diode lasers represent a significant risk to ocular tissues if accidental exposure occurs [4].

The eye is particularly vulnerable to laser-induced damage because of its natural ability to focus light on the retina, amplifying the energy density of any incoming beam [5]. The retina, particularly the macula, is at risk from visible and near-infrared lasers, as melanin and hemoglobin in the retinal pigment epithelium (RPE) and choroid strongly absorb energy [6]. Diode lasers can cause photothermal injury, leading to coagulative necrosis of the RPE and photoreceptor layers, resulting in irreversible retinal damage [7]. Depending on the energy and exposure duration, injuries can range from mild pigmentary changes to severe complications, such as macular holes, retinal scarring, or vision loss [8].

Accidental laser-induced ocular injuries are rare but more common in non-medical environments, such as beauty centers, where operators often lack formal training in laser safety [9]. Improper handling of laser devices, absence of protective eyewear, and accidental activation of laser beams can result in severe, vision-threatening injuries with long-term implications for visual function and quality of life. Delayed access to specialized ophthalmological care in such settings may further worsen outcomes.

Advanced imaging, particularly optical coherence tomography (OCT), is essential for assessing the extent of retinal damage, including macular holes, subretinal fluid, or retinal thinning, and for guiding treatment decisions [10].

In cases of full-thickness macular holes (FTMH) caused by laser injuries, surgical intervention with pars plana vitrectomy (PPV) and internal limiting membrane (ILM) peeling is often required to restore the anatomical integrity of the retina and optimize visual outcomes [11, 12]. However, the prognosis for visual recovery remains dependent on the extent of damage to the photoreceptor and RPE layers, as these are critical for central vision.

In this report, we present the case of a 24-year-old woman who sustained a FTMH with profound visual loss due to accidental exposure to a diode laser during a hair removal procedure. The case highlights the devastating consequences of inadequate laser safety protocols, the critical role of OCT in diagnosing and managing retinal injuries, and the importance of timely surgical intervention. This case also underscores the need for stricter regulations and mandatory training for laser operators in non-medical settings to prevent such injuries.

## Case report

A 24-year-old healthy woman presented to the ophthalmology emergency department with complaints of severe right eye pain, redness, and photophobia following an accidental laser exposure. The incident occurred during a routine diode laser hair removal session at a beauty center. According to the patient and accompanying staff members, the laser operator was cleaning the laser probe when an unexpected discharge from the laser device occurred, striking the patient directly in the right eye. The patient reported an immediate onset of intense ocular pain, accompanied by blurred vision and persistent tearing.

The staff at the beauty center promptly contacted emergency medical services (EMS). Upon arrival, the EMS team noted conjunctival erythema and significant discomfort in the patient’s right eye. The patient was transported to a tertiary care hospital with a dedicated ophthalmology emergency ward for further evaluation and management.

On arrival at the emergency department, the patient was alert and oriented, with stable vital signs. She endorsed no prior history of ocular disease, refractive errors, or laser exposure. The patient underwent a comprehensive ophthalmological examination following accidental diode laser exposure to the right eye. The most significant finding was a full-thickness macular hole (FTMH) in the right eye, which was associated with profound visual impairment. The left eye remained entirely normal.

Visual acuity in the right eye was reduced to the level of hand motion (HM), while the left eye retained normal vision at 20/20. External examination showed mild conjunctival injection in the right eye, with no abnormalities in the eyelids, cornea, anterior chamber, iris, or lens bilaterally. Fundoscopy of the right eye revealed a well-demarcated FTMH in the central macula, consistent with laser-induced photothermal injury. This was likely caused by the absorption of the diode laser’s energy by the retinal pigment epithelium (RPE), resulting in damage to the overlying photoreceptor layers.

The optic disc, retinal vessels, and peripheral retina were normal in both eyes. Intraocular pressure was 14 mmHg in the right eye and 13 mmHg in the left, both within normal limits.

Optical coherence tomography (OCT) confirmed the presence of the FTMH in the right eye, showing complete disruption of the foveal retinal layers with cystic changes at the margins of the hole. There was no evidence of epiretinal membrane (ERM) or vitreomacular traction (VMT). The macular architecture of the left eye was entirely normal.

**Fig. 1:**
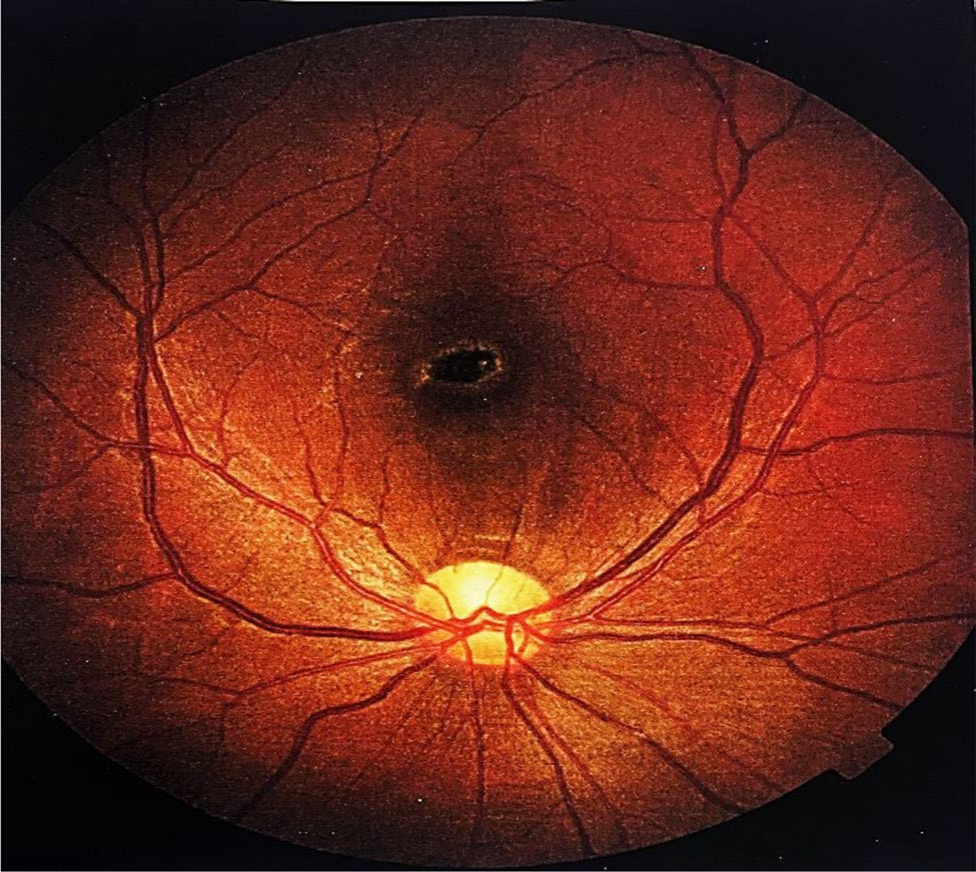
Full-Thickness Macular Hole in the Right Eye. A full-thickness macular hole (FTMH) visible in the central macula, characterized by a well-demarcated defect in the retinal layers. The edges of the macular hole show cystic changes, indicative of intraretinal edema

The findings of this case highlight the significant retinal damage caused by accidental laser exposure, with the FTMH being the most severe consequence, resulting in profound visual impairment in the affected eye. (Fig. 1).

The patient underwent a detailed evaluation and management plan following the accidental diode laser exposure to the right eye, which resulted in a FTMH and profound visual impairment. Optical coherence tomography (OCT) was performed to confirm the diagnosis and assess the extent of retinal damage. The OCT findings FTMH in the right eye, characterized by a complete disruption of the retinal layers at the fovea. The inner retinal layers were retracted, and there was a loss of continuity in the outer retinal layers, including the ellipsoid zone and retinal pigment epithelium (RPE). The central macular thickness was significantly reduced, and the edges of the hole demonstrated cystic changes, indicative of intraretinal edema. There was no evidence of epiretinal membrane (ERM) or vitreomacular traction (VMT), suggesting that the macular hole was primarily caused by the photothermal injury from the diode laser. The left eye showed normal macular architecture on OCT, with intact retinal layers and no abnormalities.

Given the severity of the findings and the profound reduction in visual acuity to the level of hand motion in the right eye, the patient was counseled regarding the need for surgical intervention. The patient underwent pars plana vitrectomy (PPV) with internal limiting membrane (ILM) peeling and gas tamponade to address the full-thickness macular hole (FTMH) and restore retinal integrity. She was counseled on the procedure’s risks, benefits, and guarded visual prognosis due to extensive photoreceptor and retinal pigment epithelium (RPE) damage caused by the laser injury.

In addition to surgical planning, the patient was started on topical corticosteroids (e.g., prednisolone acetate 1%) to reduce any residual inflammation and preservative-free artificial tears to alleviate ocular surface discomfort. Oral antioxidants, including lutein and zeaxanthin, were prescribed to support retinal health and potentially aid in the healing process. The patient was instructed to avoid any further exposure to laser devices and to wear protective eyewear in environments where laser use is possible.

In cases of laser-induced macular holes, the prognosis depends on the extent of retinal damage and the timing of surgical intervention. Early surgical repair is associated with higher rates of anatomical closure and better visual outcomes. However, in this case, the profound reduction in visual acuity and the OCT findings of significant disruption to the outer retinal layers suggest that the visual prognosis may be guarded. The patient was provided with realistic expectations regarding the potential for visual recovery and the importance of adherence to postoperative care.

## Discussion

The case presented in this report illustrates the severe consequences of accidental diode laser exposure to the eye, resulting in a FTMH and profound vision loss. While diode lasers are considered safe when used appropriately, improper handling and inadequate safety measures, such as the absence of protective eyewear, can lead to devastating ocular injuries.

Laser-induced ocular injuries can occur through three primary mechanisms: photothermal, photochemical, and photo disruptive damage, depending on the laser’s wavelength, pulse duration, and energy delivered. Photothermal damage, the most common mechanism, results from heat generation in pigmented tissues, leading to protein denaturation and tissue necrosis. Photochemical damage occurs due to prolonged exposure to low-energy ultraviolet or blue light, producing reactive oxygen species and molecular damage. Photo disruptive damage, associated with high-energy pulsed lasers, causes mechanical disruption of tissues through plasma formation and shockwave propagation [13, 14].

Different laser types can cause macular holes and other retinal injuries under specific scenarios:

- **Diode lasers (800–810 nm)**: Commonly used in dermatological and cosmetic procedures, diode lasers can cause photothermal damage to the RPE and photoreceptor layers, leading to FTMH, as presented in this case [7, 8].
- **Nd: YAG lasers (1064 nm/532 nm)**: Used in ophthalmology for posterior capsulotomy or iridotomy, Nd: YAG lasers can induce photo disruptive damage to retinal structures when misdirected [13].
- **Laser pointers (650–532 nm)**: Recreational laser pointers, particularly high-powered models, can cause macular injury through photothermal and photo disruptive effects, especially in children and untrained users [15].
- **Intense Pulsed Light (IPL)**: Frequently used in cosmetic treatments, IPL can cause thermal injury to the retina, especially when protective eyewear is not utilized [16].
- **Alexandrite lasers (755 nm)**: Commonly employed in dermatology, alexandrite lasers can cause retinal damage through photothermal mechanisms when used near the periorbital region without appropriate eye protection [17].

The risk of laser-induced ocular damage can be minimized by adhering to strict safety protocols, including proper training of operators, use of wavelength-specific protective eyewear, and avoiding laser activation in unprotected environments. Table 1 summarizes the mechanisms, scenarios, and preventive strategies for different laser types.

**Table 1:** Laser types, mechanisms of injury, and preventive strategies

Laser type	Wavelength	Mechanism of injury	Common scenarios	Preventive strategies
Diode Laser	800_810 nm	Photothermal	Cosmetic procedures (e.g., hair removal)	Operator training, protective eyewear, avoid accidental activation
Nd: YAG Laser	1064 nm, 532 nm	Photo disruptive	Ophthalmic procedures (e.g., capsulotomy, iridotomy)	Proper alignment, training, protective eyewear
Laser Pointer	532_650 nm	Photothermal, Photo disruptive	Recreational use, especially in children	Regulation of laser power, education on safe usage
Intense Pulsed Light (IPL)	Broad spectrum (515–1200 nm)	Photothermal	Cosmetic treatments (e.g., facial rejuvenation)	Wavelength-specific protective eyewear, operator training
Alexandrite Laser	755 nm	Photothermal	Dermatologic procedures near periorbital areas	Eye shields, avoidance of treatment near eyes

As previously mentioned, Photothermal damage, the most common mechanism, results from localized heat buildup, causing protein denaturation, tissue necrosis, and scarring. In the presented case, accidental diode laser exposure caused a FTMH and significant vision loss due to thermal damage, highlighting the severe consequences of inadequate safety measures [14, 15]. The resulting damage led to the formation of a macular hole, which is a rare but vision-threatening complication of laser-induced retinal injury [8].

Advanced imaging, particularly optical coherence tomography (OCT), played a critical role in this case by enabling high-resolution visualization of retinal architecture and confirming the presence of a FTMH [18]. OCT findings, including disruption of the foveal retinal layers and cystic changes at the margins of the hole, were consistent with the diagnosis and offered valuable insights into the extent of retinal damage [19]. This aligns with existing literature, which highlights OCT as an essential diagnostic tool for assessing laser-induced retinal injuries and guiding management decisions [20, 21].

Spontaneous closure of FTMH has occasionally been reported in idiopathic cases; however, the likelihood of spontaneous closure in laser-induced macular holes is exceedingly rare [22]. This is due to the extensive photothermal damage to the RPE and photoreceptor layers, which significantly reduces the potential for natural anatomical repair [23]. While the incidence of spontaneous closure in laser-induced macular holes is not well-documented, the available evidence suggests that severe retinal damage in such cases makes spontaneous resolution highly unlikely [22].

Given this, the treatment of laser-induced macular holes typically involves PPV with ILM peeling and gas tamponade. This approach is widely regarded as the gold standard for achieving anatomical closure of macular holes, regardless of their etiology [24, 25]. In cases where surgery is not immediately feasible, a short observation period of 4–6 weeks with close monitoring using OCT may be considered. However, prolonged delays in surgical management could negatively impact both anatomical and functional outcomes, as demonstrated by the guarded visual prognosis in the present case [26, 27].

In this case, PPV with ILM peeling and gas tamponade successfully achieved anatomical closure of the macular hole. However, visual recovery was limited due to the extent of RPE and photoreceptor damage. This outcome underscores the importance of laser safety protocols to prevent such injuries, as the potential for functional recovery remains dependent on the severity of initial damage [28, 29].

A review identified 119 cases of ocular injuries associated with dermatologic laser treatments, with 60 involving direct damage to ocular structures, primarily due to inadequate eye protection (73%). Laser hair removal of the face was the most common procedure (58%), and nearly all injuries (98%) were specific to the laser’s wavelength and chromophore affinity. Most cases were preventable, highlighting the critical need for proper safety measures [7].

Maganti et al. reported the first case of macular hole formation following intense pulsed light (IPL) therapy for hair removal in a 68-year-old woman who experienced blurry vision and a FTMH after accidental IPL exposure without proper eye protection. Like the case presented in this study, the mechanism of injury was attributed to thermal damage at the level of the RPE, highlighting the vulnerability of retinal tissue to light-based cosmetic treatments. While Maganti et al. focused on IPL-induced injury, the current case involves diode laser-induced FTMH, emphasizing the risks associated with different laser technologies and the shared importance of strict adherence to safety protocols, including the use of wavelength-specific protective eyewear, to prevent potentially vision-threatening ocular injuries [16].

In another study, Hammes et al. re ported a 33-year-old woman with a port-wine stain underwent multiple laser treatments, including a session with a 755-nm alexandrite laser in the periorbital region, during which protective eye shields were not used. Postoperatively, she developed photophobia, blurred vision, scleral inflammation, posterior synechia, and an irregular, non-reactive left pupil. Despite treatment, the pupil remained irregular and dilated at three months. Eleven months after the procedure, there was significant improvement in pupil motility due to constant topical treatment with diclofenac sodium; however, photophobia persisted, and the patient reported vision distortion during movement [17].

The case presented in this study underscores the critical importance of adhering to stringent laser safety protocols to prevent severe, vision-threatening ocular injuries during dermatologic procedures. Numerous studies have identified gaps in safety practices, including the absence of mandatory reporting, inadequate training, and a lack of regulation for non-physician operators, all of which increase the risk of preventable injuries [30–32]. The American National Standards Institute (ANSI) guidelines highlight the need for wavelength-specific protective eyewear and detailed safety protocols to mitigate risks, particularly in procedures involving the periorbital area [30, 31]. Furthermore, Kalashnikova et al. emphasized the role of operator error, neglect of treatment protocols, and inadequate safety measures as primary causes of complications, stressing the importance of patient selection, device maintenance, and adherence to treatment protocols [32]. Glover and Richer also noted that 73% of ocular injuries from laser procedures could have been avoided with proper eye protection, further demonstrating the necessity of rigorous safety measures and standardized operator training [30]. This case reinforces the need for comprehensive safety guidelines, regular audits, and improved reporting systems within clinical and non-medical settings to reduce the incidence of preventable laser-induced ocular injuries.

## Conclusion

This case highlights the severe and vision-threatening complications that can occur from accidental diode laser exposure, particularly in non-medical environments where safety protocols are often disregarded. The development of a FTMH in this patient underscores the critical importance of proper laser safety measures, including the mandatory use of wavelength-specific protective eyewear and operator training. While timely surgical intervention successfully restored retinal integrity, the extent of retinal damage limited visual recovery, emphasizing the need for prevention over treatment.

This report reinforces the necessity of stricter regulatory oversight, standardized safety protocols, and public awareness to minimize the risk of laser-induced ocular injuries and safeguard patients’ vision in both medical and non-medical settings.

## Data Availability

The datasets used or analyzed during the current study are available from the corresponding author upon reasonable request. This includes anonymized patient data to ensure confidentiality while allowing for scientific scrutiny and verification of results.

## Declarations

### Limitations

We acknowledge the absence of OCT images and long-term follow-up data as limitations of this case report. Unfortunately, the OCT images were not preserved in the patient’s records, and the patient was lost to follow-up after the initial surgical intervention. While these limitations restrict our ability to provide imaging evidence and long-term outcomes, the case remains significant due to the rare presentation of a full-thickness macular hole caused by diode laser exposure in a non-medical setting. This report highlights the critical need for improved safety protocols and regulations in such environments.

## References

[1] Durmuş Ho. Fundamentals of Medical Laser Technology and its Various Medical Applications. Current Research in Medicine and Health Sciences-2024:73.

[2] Anderson RR, Parrish JA. Selective photothermolysis: precise microsurgery by selective absorption of pulsed radiation. Science. 1983;220(4596):524–7. 6836297 10.1126/science.6836297

[3] Lanigan S. Therapeutic applications: Dermatology—Selective photothermolysis. Handbook of laser technology and applications. CRC; 2021. pp. 267–72.

[4] Fayne RA, Perper M, Eber AE, Aldahan AS, Nouri K. Laser and light treatments for hair reduction in Fitzpatrick skin types IV–VI: A comprehensive review of the literature. Am J Clin Dermatol. 2018;19:237–52. 28791605 10.1007/s40257-017-0316-7

[5] Barkana Y, Belkin M. Laser eye injuries. Surv Ophthalmol. 2000;44(6):459–78. 10906379 10.1016/s0039-6257(00)00112-0

[6] Chen S, Lu C, Hu X, Zhou D. A case of accidental retinal injury by cosmetic laser. Eye. 2014;28(7):906–7. 24763243 10.1038/eye.2014.81 PMC4094815

[7] Flegel L, Kherani F, Richer V. Review of eye injuries associated with dermatologic laser treatment. Dermatol Surg. 2022;48(5):545–50. 35333214 10.1097/DSS.0000000000003427

[8] Mainster MA. Retinal laser accidents: mechanisms and management. J Laser Appl. 2000;12(1):3–9.

[9] Yan MK, Kocak E, Yoong K, Kam JK. Ocular injuries resulting from commercial cosmetic procedures. Clin Experimental Optometry. 2020;103(4):430–3. 10.1111/cxo.12952 31382317

[10] Murthy R, Haji S, Sambhav K, Grover S, Chalam K. Clinical applications of spectral domain optical coherence tomography in retinal diseases. Biomedical J. 2016;39(2):107–20. 27372166 10.1016/j.bj.2016.04.003 PMC6138795

[11] Kandari JA, Raizada S, Razzak AA. Accidental laser injury to the eye. Ophthalmic Surg Lasers Imaging Retina. 2010;41(3):1–5. 10.3928/15428877-20100215-26 20337345

[12] Ruggeri ML, Quarta A, Marolo P, Zeppa L, Motta L, Gironi M, et al. Comparison of conventional internal limiting membrane versus Pars plana vitrectomy without peeling for small idiopathic macular hole. Int J Retina Vitreous. 2024;10(1):81. 39449027 10.1186/s40942-024-00599-5 PMC11515618

[13] Wong EWN, Lai AC-h, Lam RF, Lai FHP. Laser-induced ocular injury: a narrative review. Hong Kong J Ophthalmol. 2020;24(2).

[14] Yiu G, Itty S, Toth CA. Ocular safety of recreational lasers. JAMA Ophthalmol. 2014;132(3):245–6. 24407269 10.1001/jamaophthalmol.2013.5647

[15] Khurana AK. Comprehensive ophthalmology: with supplementary book-review of ophthalmology. JP Medical Ltd; 2015.

[16] Maganti N, Kalbag NS, Gill MK. Macular hole formation associated with intense pulsed light therapy. Retinal Cases Brief Rep. 2022;16(2):161–4. 10.1097/ICB.0000000000000947 PMC8860208 31851048

[17] Hammes S, Augustin A, Raulin C, Ockenfels H-M, Fischer E. Pupil damage after periorbital laser treatment of a port-wine stain. Arch Dermatol. 2007;143(3):392–4. 17372105 10.1001/archderm.143.3.392

[18] Schuman JS, Fujimoto JG, Duker J, Ishikawa H. Optical coherence tomography of ocular diseases: CRC; 2024.

[19] Arevalo J, Sanchez J, Costa R, Farah ME, Berrocal M, Graue-Wiechers F, et al. Optical coherence tomography characteristics of full-thickness traumatic macular holes. Eye. 2008;22(11):1436–41. 17828143 10.1038/sj.eye.6702975

[20] Lains I, Wang JC, Cui Y, Katz R, Vingopoulos F, Staurenghi G, et al. Retinal applications of swept source optical coherence tomography (OCT) and optical coherence tomography angiography (OCTA). Prog Retin Eye Res. 2021;84:100951. 33516833 10.1016/j.preteyeres.2021.100951

[21] Mrejen S, Spaide RF. Optical coherence tomography: imaging of the choroid and beyond. Surv Ophthalmol. 2013;58(5):387–429. 23916620 10.1016/j.survophthal.2012.12.001

[22] Zvizdic D, Nisic F, Ljaljevic S, Zvizdic Z, Vranic S. Spontaneous closure of the large idiopathic full-thickness macular hole. 2023. 10.1016/j.asjsur.2023.08.052 37591753

[23] Keshet Y, Weseley PE, Ceisler EJ, Ngo WK, Salcedo A, Walia J et al. The evolution of full thickness macular hole after short exposure to high powered Hand-held laser pointer. Retinal Cases Brief Rep. 2022:101097. 10.1097/ICB.0000000000001374 36730459

[24] Ghoraba HH, Leila M, Ghoraba H, Heikal MA, Elgemai EEM. Comparative study between Pars plana vitrectomy with internal limiting membrane Peel and Pars plana vitrectomy with internal limiting membrane flap technique for management of traumatic full thickness macular holes. J Ophthalmol. 2019;2019(1):1959082. 31143468 10.1155/2019/1959082 PMC6501415

[25] Figueroa MS, Govetto A, Steel DH, Sebag J, Virgili G, Hubschman JP. Pars plana vitrectomy for the treatment of tractional and degenerative lamellar macular holes: functional and anatomical results. Retina. 2019;39(11):2090–8. 30312255 10.1097/IAE.0000000000002326

[26] Rush RB, Rush SW. Pars plana vitrectomy with internal limiting membrane peeling for treatment-naïve diabetic macular edema: a prospective, uncontrolled pilot study. Clin Ophthalmol. 2021:2619–24. 10.2147/OPTH.S320214 PMC8232852 34188440

[27] Chen Y, Lu N, Li J, Yu J, Yu Y, Shi X. Results of Pars plana vitrectomy with peeling of the inner limiting membrane in patients with laser-induced macular hole. Zhonghua Yi Xue Za Zhi. 2018;98(48):3941–5. 30669799 10.3760/cma.j.issn.0376-2491.2018.48.007

[28] Ittarat M, Somkijrungroj T, Chansangpetch S, Pongsachareonnont P. Literature review of surgical treatment in idiopathic full-thickness macular hole. Clin Ophthalmol. 2020:2171–83. 10.2147/OPTH.S262877 PMC7398756 32801628

[29] Cornish KS, Lois N, Scott NW, Burr J, Cook J, Boachie C, et al. Vitrectomy with internal limiting membrane peeling versus no peeling for idiopathic full-thickness macular hole. Ophthalmology. 2014;121(3):649–55. 24314837 10.1016/j.ophtha.2013.10.020

[30] Glover C, Richer V. Preventing eye injuries from light and laser-based dermatologic procedures: a practical review. J Cutan Med Surg. 2023;27(5):509–15. 37533142 10.1177/12034754231191064 PMC10616986

[31] Sayed MS, Ko MJ, Ko AC, Lee WW. Ocular damage secondary to lights and lasers: how to avoid and treat if necessary. World J Ophthalmol. 2014;4(1):1–6.

[32] Kalashnikova NG, Jafferany M, Lotti T. Management and prevention of laser complications in aesthetic medicine: an analysis of the etiological factors. Dermatol Ther. 2021;34(1):e14373. 33029827 10.1111/dth.14373

